# Proteomic alteration of endometrial tissues during secretion in polycystic ovary syndrome may affect endometrial receptivity

**DOI:** 10.1186/s12014-022-09353-1

**Published:** 2022-05-28

**Authors:** Jun Li, Xiaohua Jiang, Caihua Li, Huihui Che, Lin Ling, Zhaolian Wei

**Affiliations:** 1 Department of Obstetrics and Gynecology, The Second Affiliated Hospital of Anhui Medical University, Hefei, 230022 China; 2 Department of Obstetrics and Gynecology, Reproductive Medicine Center, The First Affiliated Hospital of Anhui Medical University, Hefei, 230022 China; 3 Anhui Province Key Laboratory of Reproductive Health and Genetics, Anhui Medical University, Hefei, 230022 China; 4 Anhui Provincial Engineering Technology Research Center for Biopreservation and Artificial Organs, Hefei, 230022 China

**Keywords:** Endometrial receptivity, Endometrium, PCOS, Proteomics

## Abstract

Embryo implantation is a complex developmental process that requires coordinated interactions among the embryo, endometrium, and the microenvironment of endometrium factors. Even though the impaired endometrial receptivity of patients with polycystic ovary syndrome (PCOS) is known, understanding of endometrial receptivity is limited. A proteomics study in three patients with PCOS and 3 fertile women was performed to understand the impaired endometrial receptivity in patients with PCOS during luteal phases. Through isobaric tags for relative and absolute quantitation (iTRAQ) analyses, we identified 232 unique proteins involved in the metabolism, inflammation, and cell adhesion molecules. Finally, our results suggested that energy metabolism can affect embryo implantation, whereas inflammation and cell adhesion molecules can affect both endometrial conversion and receptivity. Our results showed that endometrial receptive damage in patients with PCOS is not a single factor. It is caused by many proteins, pathways, systems, and abnormalities, which interact with each other and make endometrial receptive research more difficult.

**Supplementary Information:**

The online version contains supplementary material available at 10.1186/s12014-022-09353-1.

## Introduction

Polycystic ovary syndrome (PCOS) is the most common endocrine disorder among women of reproductive age and perplexes researchers and doctors globally [1]. Even though many researchers focus on the pathophysiology of PCOS, the etiology underlying PCOS is still unknown. Many present studies mainly focused on improving clinical symptoms, such as insulin resistance, obesity, metabolic derangements, and increase in androgen, to achieve successful conceiving, reduce pregnancy-related complications, and enhance pregnancy outcomes [2, 3]. Ovulation disorders were previously considered the main cause of infertility in patients with PCOS. The pregnancy rates are still low in patients with PCOS and the high risk of biochemical abortion after ovulation disorders have been reduced. Many factors may lead to this situation, and impaired endometrial receptivity could be a responsible reason for adverse pregnancy outcomes in patients with PCOS. Unfortunately, only a few studies have elucidated the molecular mechanisms underlying impaired endometrial receptivity. Some important proteins involved in embryo implantation, such as forkhead box protein O1 (FOXO1), homeobox A10 (HOXA10), insulin-like growth factor-binding protein 1 (IGFBP-1), and inhibiting insulin growth factor 1 (IGF-1) are known to be abnormal in patients with PCOS compared with healthy individuals [4]. Single protein changes do not reflect the function of the endometrial microenvironment due to protein–protein interactions; therefore, the ongoing studies have increasingly focused on proteomic analyses. Proteomics-based analyses are not limited by previous information on the problem and can help discover the potential advantage of revealing novel associations with unexpected molecules that can lead to new mechanistic explanations for impaired endometrial implantation.

In the present years, proteomics analyses have been used to elucidate the potential mechanisms underlying adverse pregnancy outcomes in patients with PCOS. To the best of our knowledge, no research has been performed on the secretory endometrial proteome in patients with PCOS to date. To elucidate the molecular basis underlying infertility related to endometrium implantation in patients with PCOS, we compared the secretory endometrial proteomic profile of patients with PCOS with that of healthy fertile women using isobaric tags for relative and absolute quantitation (iTRAQ).

## Materials and methods

### Clinical sample preparation methods

The endometrial tissues were obtained from 3 patients with PCOS and 3 healthy volunteers who already had children. The patients with PCOS took letrozole on the 3rd day of menstruation; their ovulation was continuously monitored, starting from the 10th day of menstruation; and the endometrium was obtained on the 5th day of ovulation.

**Fig. 1:**
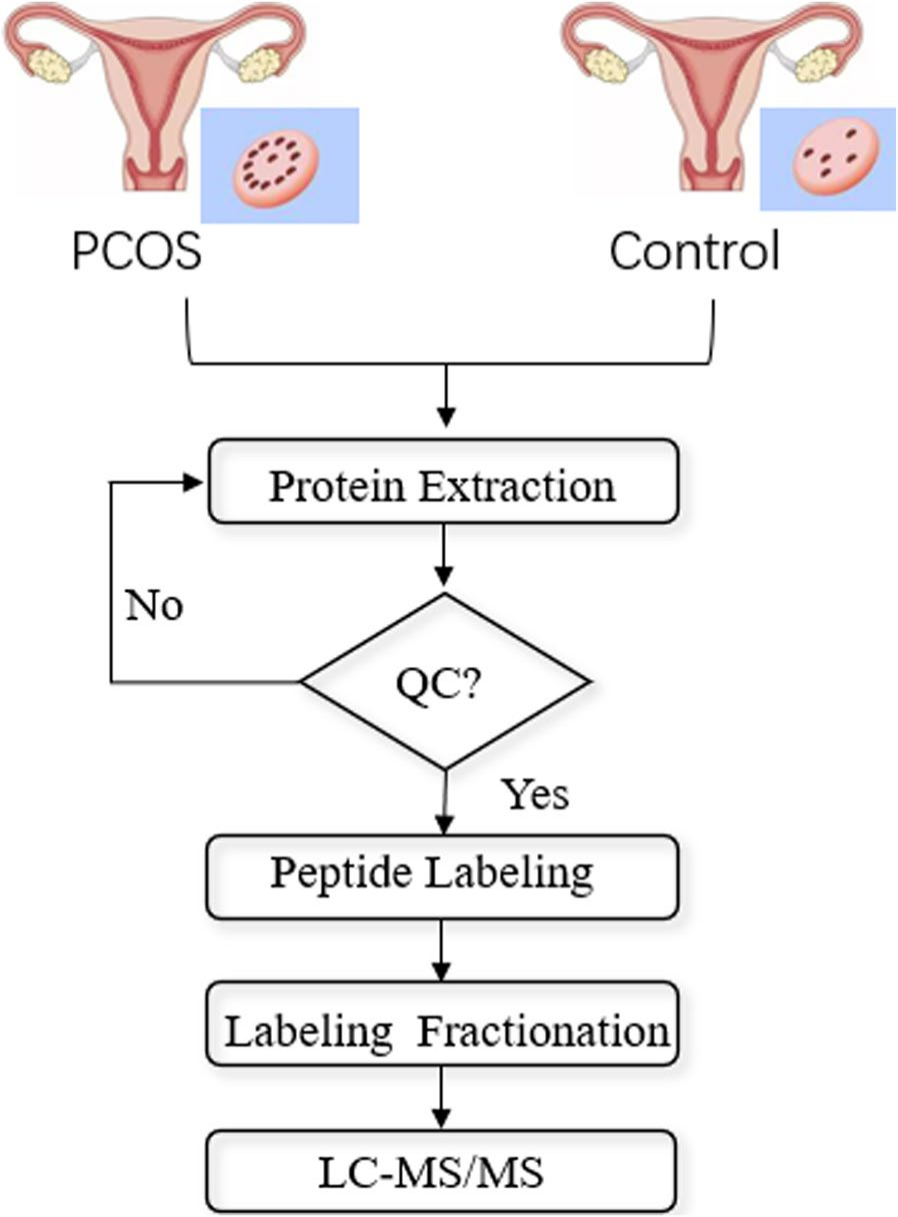
Experimental procedures. This figure shows the main procedures of the experiment of iTRAQ quantitative proteomics

These patients were also screened for their glucose metabolism and endocrine normality through serum determinations of the levels of follicle-stimulating hormone (FSH), luteinizing hormone (LH), estradiol, glucose, and insulin on day 3 of the menstrual cycle. No participants demonstrated any evidence of chromosomal abnormality, pathological uterine disorder, or endometrial hyperplasia. None of the patients had used oral contraception or had undergone hormonal therapy during the past 3 months. The diagnosis of PCOS was made in accordance with the 2003 Rotterdam criteria, which included any two or all three of the following features: (1) oligo-/anovulation; (2) clinical or biochemical signs of hyperandrogenism; and (3) polycystic ovary morphology on ultrasound examination [5]. The main demographic characteristics of the patient and the control groups are summarized in Table 1. The results for the PCOS and control groups did not differ in terms of age, body mass index (BMI), FSH, LH, and testosterone, albeit it differed for the levels of insulin and glucose. Each biopsy was dry frozen at − 80 °C for protein extraction. The patients were recruited at the Reproductive Medicine Center, Department of Obstetrics and Gynecology, The First Affiliated Hospital of Anhui Medical University, approved by the Institutional Ethics Committee (No: 20170609). All patients provided their informed consent prior to their participation in the study. Figure 1 displays the basic principle of iTRAQ quantitative proteomics and the main steps involved in the quantitative techniques.

**Table 1:** Demographic characteristics of PCOS and control subjects

	PCOS (n = 3)	Control(n = 3)	*P*
Age years	29.67 ± 0.58	30.67 ± 1.15	0.25
BMI kg/m^2^	24.96 ± 2.15	21.10 ± 1.47	0.06
FSH IU/L	5.24 ± 1.30	6.44 ± 1.28	0.32
LH IU/L	6.43 ± 2.62	3.41 ± 2.23	0.20
Testosterone nmol/L	1.41 ± 0.26	1.11 ± 0.20	0.35
Insulin mU/L	12.14 ± 2.01	6.24 ± 0.92	0.01
Glucose mmol/L	5.80 ± 0.31	5.23 ± 0.19	0.05

### Protein extraction

We used the lysis buffer 3 (8 M urea, TEAB or 40 mM Tris–HCl with 1 mM PMSF, 2 mM EDTA and 10 mM DTT; pH 8.5) and two magnetic beads to extract the proteins. Then, we removed the mixtures into a tissue lyser for 2 min at 50 Hz to release the proteins. Next, the supernatant was transferred into a new tube after centrifugation at 25,000×*g* at 4 °C for 20 min, reduced with 10-mM dithiothreitol (DTT) at 56 °C for 1 h, and alkylated with 55-mM iodoacetamide (IAM) in the dark at room temperature for 45 min. Following centrifugation, the supernatant containing the proteins was quantified by Bradford assay.

### QC of protein extraction

#### Protein quantitation by Bradford assay

First, we added 0, 2, 4, 6, 8, 10, 12, 14, 16, and 18 μL of the BSA solution, separately, into a 96-well plate, and to the corresponding wells, we added 20, 18, 16, 14, 12, 10, 8, 6, 4, and 2 μL of pure water, separately. Meanwhile, we prepared serial dilutions (20 μL/well) of the unknown sample for enumeration. Next, we added 180 μL of Coomassie blue to each well and mixed the contents of each well. The absorbance of each standard and sample well were read at 595 nm. Each sample had at least two duplicates. Then, the absorbance of the standards vs. their concentration was plotted. Finally, we calculated the extinction coefficient and the concentrations of the unknown samples.

### Protein digestion

The protein solution (100 μg) containing 8 M urea was diluted 4 times with 100 mM TEAB. We then applied trypsin gold (Promega, Madison, WI, USA) to digest the proteins (protein: trypsin = 40:1) at 37 °C overnight. Next, we used the Strata X C18 column (Phenomenex) and vacuum-dried the specimens to desalt the peptides in accordance with the manufacturer's protocol.

### Peptide labeling

We dissolved the peptides in 30 μL of 0.5 M TEAB by vortexing. Then, the iTRAQ labeling reagents were recovered to the ambient temperature and transferred and combined with the appropriate samples. Immediately before labeling the peptides, IBT precursors were treated with an equal molar ratio of TSTU (1,1,3,3-tetramethyl-O-(N-succinimidyl) uronium tetrafluoroborate) sourced from TCI (Shanghai, China) in isopropanol to a final concentration of 25 μg/μL and incubated at room temperature for 10 min. The activated IBT was mixed with a certain amount of peptides dissolved in 0.2 M triethylammonium bicarbonate (TEAB). In the labeling reaction, the isopropanol concentration was maintained at > 75%, and the labeling process was stopped by adding trifluoroacetic acid (TFA) at the end of the incubation period at the ambient temperature for 2 h. Then, we combined and desalted the labeled peptides on the Strata X C18 column and vacuum-dried them as per the manufacturer’s protocol.

### Peptide fractionation

We separated the peptides through the Shimadzu LC-20AB HPLC Pump System coupled with a high-pH RP column. Next, we reconstituted the peptides with buffer A (5% ACN, 95% H_2_O, adjusted the pH to 9.8 with ammonia) to 2 mL and loaded them onto a column (5 μm, 20 cm × 180 μm; Gemini C18) containing 5-μm particles (Phenomenex). Then, we separated the peptides at the flow rate of 1 mL/min with a gradient of 5% buffer B (5% H_2_O, 95% ACN, adjusted pH to 9.8 with ammonia) for 10 min, 5–35% buffer B for 40 min, and 35–95% buffer B for 1 min. Then, the system was maintained in 95% buffer B for another 3 min and decreased to 5% within 1 min before equilibration with 5% buffer B for 10 min. Next, we monitored the elution by measuring the absorbance at 214 nm and collected the fractions every minute. Finally, we divided the eluted peptides into 20 fractions and vacuum-dried them for further analyses.

### HPLC

First, each fraction was resuspended in buffer A (2% ACN, 0.1% FA) and centrifuged at 20,000×*g* for 10 min. Then, the supernatant was loaded on the Thermo Scientific™ UltiMate™ 3000 UHPLC system equipped with a trap and an analytical column. We loaded the samples on the trap column (PEPMAP 100 C18 5UM 0.3X5MM 5PK) at 5 μL/min for 8 min and eluted it into the homemade nanocapillary C18 column (ID 75 μm × 25 cm, 3-μm particles) with a 300 nL/min flow rate. The gradient of buffer B (98% ACN, 0.1% FA) was raised from 5 to 25% in 40 min, raised to 35% in 5 min, followed by a 2-min linear gradient to 80%, maintained at 80% B for another 2 min, returned to 5% in 1 min, and then equilibrated for 6 min.

### Mass spectrometer detection

We subjected the peptides separated from nanoHPLC to tandem mass spectrometry Q EXACTIVE HF X (Thermo Fisher Scientific, San Jose, CA) for data-dependent acquisition (DDA) detection by nanoelectrospray ionization. The relevant parameters of the MS analysis were as follows: precursor scan range: 350–1500 m/z at the resolution of 60,000 in Orbitrap; electrospray voltage: 2.0 kV; MS/MS fragment scan range: in HCD mode with a 100 m/z scan, resolution at 15,000; normalized collision energy setting: 30%; dynamic exclusion time: 30 s; automatic gain control (AGC) for full MS target and MS2 target: 3e6 and 1e5, respectively; the number of MS/MS scans following one MS scan: 20 most abundant precursor ions above a threshold ion count of 10,000.

### Protein quantification

We used an automated software called IQuant to quantitatively analyze the labeled peptides with isobaric tags. This software integrates the Mascot Percolator [6] to provide reliable significance measurements. To assess the confidence of peptides, the PSMs were prefiltered at 1% PSM-level FDR. Then, based on the “simple principle” (the parsimony principle), the identified peptide sequences were assembled into a set of confident proteins. To control the rate of false positives at the protein level, a protein FDR of 1%, which is based on the selected protein FDR strategy [7], was estimated after protein inference (protein-level FDR ≤ 0.01). The process of protein quantification comprised the following steps: protein identification, tag impurity correction, data normalization, missing value imputation, protein ratio calculation, statistical analysis, and result presentation [7]. Data normalization: We selected variance stabilization normalization (VSN) [8, 9] as our preferred normalization strategy. Protein ratio calculation: nonunique peptides and outlier peptide ratios were removed prior to their quantification [10]. The weight approach proposed elsewhere [11] was employed to evaluate the ratios of protein quantity based on the reporter ion intensities. Statistical analysis: Permutation tests were widely applied in the fields of microarray and RNA-Seq data analysis [12, 13]. To estimate the statistical significance of the protein quantitative ratios, IQuant adopted the permutation test, a nonparametric approach, as reported by Nguyen et al. [14]. For each protein, IQuant provided a significance evaluation that was corrected for multiple hypothesis testing by the Benjamini–Hochberg method [15].

## Results

### Altered levels of proteins in the endometrium of women with PCOS

**Fig. 2:**
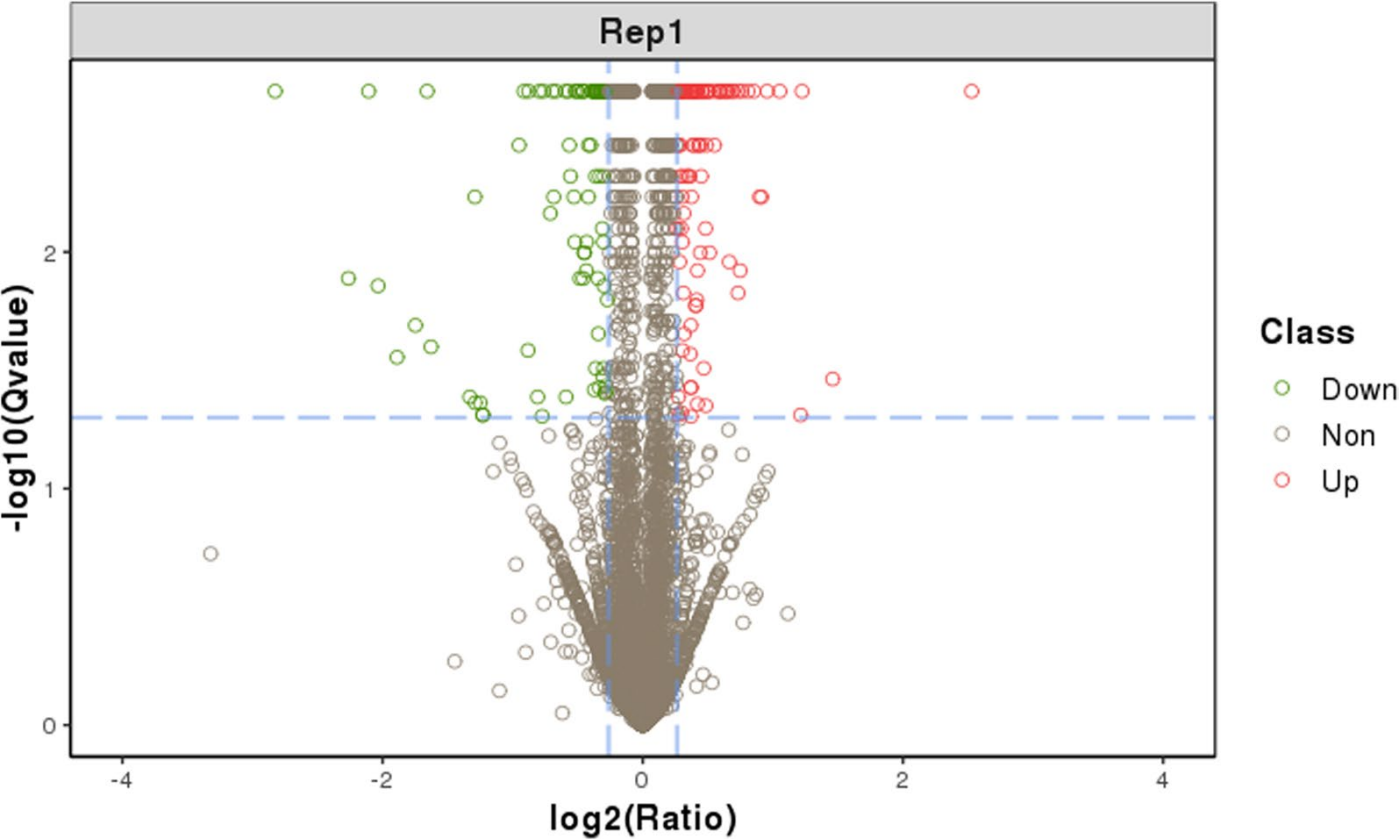
Volcano of differentially expressed proteins. This plot depicts volcano plot of log2 fold-change (x-axis) versus -log10 Q value (y-axis, representing the probability that the protein is differentially expressed). Q value < 0.05 and Fold change > 1.2 are set as the significant threshold for differentially expression. The red and green dots indicate points-of-interest that display both large-magnitude fold-changes as well as high statistical significance. Dots in red mean significant up-regulated proteins which passed screening threshold. Dots in green mean significant down-regulated proteins which passed screening threshold. And gray dots are non-significant differentially expressed protein

We quantitatively identified 6524 proteins in samples from the PCOS group and the control group. We used *CV* to evaluate the reproducibility. *CV* is defined as the ratio of the standard deviation (SD) and the mean. Lower *CV* indicates better reproducibility. The mean *CV* (0.12) showed that the proteins identified in this study have good reproducibility. (Additional file 1: Fig. S1). Proteins with a 1.2-fold change and Q value less than 0.05 were determined as differentially expressed proteins (DEPs) in a single replicate. Compared with the control group, 232 proteins showed significant changes in their levels in the PCOS group. Of these, 108 proteins were increased and 124 proteins were decreased. The list of significantly regulated proteins along with their log 2 changes, corresponding p-values, and relevant biological processes are shown in Fig. 2 and Table 2.

**Table 2:** List of significantly regulated proteins in PCOS and control groups

No.	Protein_ID	Description	*P*	Mean_Ratio_treated-VS-control
1	sp|Q7Z6B0|CCD91_HUMAN	Coiled-coil domain-containing protein 91 (CCDC91)	0.00	0.82
2	sp|Q8NHQ9|DDX55_HUMAN	ATP-dependent RNA helicase DDX55 (DDX55)	0.00	0.82
3	sp|Q9Y6Q1|CAN6_HUMAN	Calpain-6 (CAPN6)	0.00	0.79
4	sp|Q9NYC9|DYH9_HUMAN	Dynein heavy chain 9, axonemal (DNAH9)	0.00	0.33
5	sp|Q9BZW7|TSG10_HUMAN	Testis-specific gene 10 protein (TSGA10)	0.00	0.8
6	sp|Q9NSY0|NRBP2_HUMAN	Nuclear receptor-binding protein 2 (NRBP2)	0.02	0.8
7	sp|O60331|PI51C_HUMAN	Phosphatidylinositol 4-phosphate 5-kinase type-1 gamma (PIP5K1C)	0.03	0.7
8	sp|Q9Y4X5|ARI1_HUMAN	E3 ubiquitin-protein ligase ARIH1 (ARIH1)	0.03	0.74
9	sp|P01602|KV105_HUMAN	Immunoglobulin kappa variable 1-5 (IGKV1-5)	0.01	0.74
10	sp|Q8N6U8|GP161_HUMAN	G-protein coupled receptor 161 (GPR161)	0.04	0.83
11	sp|P05543|THBG_HUMAN	Thyroxine-binding globulin (SERPINA7)	0.02	0.82
12	sp|Q9NX55|HYPK_HUMAN	Huntingtin-interacting protein K (HYPK)	0.00	0.74
13	sp|P55058|PLTP_HUMAN	Phospholipid transfer protein (PLTP)	0.04	0.78
14	sp|O75015|FCG3B_HUMAN	Low affinity immunoglobulin gamma Fc region receptor III-B (FCGR3B)	0.04	0.82
15	sp|Q9HCJ0|TNR6C_HUMAN	Trinucleotide repeat-containing gene 6C protein (TNRC6C)	0.03	0.76
16	sp|P04439|1A03_HUMAN	HLA class I histocompatibility antigen, A-3 alpha chain (HLA-A)	0.05	0.65
17	sp|Q9H8V3|ECT2_HUMAN	Protein ECT2 (ECT2)	0.03	0.81
18	sp|O43174|CP26A_HUMAN	Cytochrome P450 26A1 (CYP26A1)	0.02	0.83
19	sp|Q9P2F6|RHG20_HUMAN	Rho GTPase-activating protein 20 (ARHGAP20)	0.00	0.75
20	sp|Q9NVQ4|FAIM1_HUMAN	Fas apoptotic inhibitory molecule 1 (FAIM)	0.00	0.71
21	sp|Q8NAN2|MIGA1_HUMAN	Mitoguardin 1 (MIGA1)	0.00	0.56
22	sp|Q8ND83|SLAI1_HUMAN	SLAIN motif-containing protein 1 (SLAIN1)	0.01	0.83
23	sp|Q9UP95|S12A4_HUMAN	Solute carrier family 12 member 4 (SLC12A4)	0.01	0.76
24	sp|Q96D05|F241B_HUMAN	Uncharacterized protein FAM241B (FAM241B)	0.00	0.77
25	sp|Q13009|TIAM1_HUMAN	T-lymphoma invasion and metastasis-inducing protein 1 (TIAM1)	0.03	0.65
26	sp|A0A0C4DH29|HV103_HUMAN	Immunoglobulin heavy variable 1-3 (IGHV1-3)	0.04	0.72
27	sp|P01597|KV139_HUMAN	Immunoglobulin kappa variable 1-39 (IGKV1-39)	0.00	0.79
28	sp|A0A075B6I0|LV861_HUMAN	Immunoglobulin lambda variable 8-61 (IGLV8-61)	0.03	0.77
29	sp|Q99969|RARR2_HUMAN	Retinoic acid receptor responder protein 2 (RARRES2)	0.02	0.72
30	sp|Q8N9N8|EIF1A_HUMAN	Probable RNA-binding protein EIF1AD (EIF1AD)	0.03	0.8
31	sp|P0DOX3|IGD_HUMAN	Immunoglobulin delta heavy chain	0.01	0.83
32	sp|Q15751|HERC1_HUMAN	Probable E3 ubiquitin-protein ligase HERC1 (HERC1)	0.00	0.81
33	sp|P62837|UB2D2_HUMAN	Ubiquitin-conjugating enzyme E2 D2 (UBE2D2)	0.00	0.81
34	sp|A0A0B4J1Y8|LV949_HUMAN	Immunoglobulin lambda variable 9-49 (IGLV9-49)	0.00	0.82
35	sp|P0DP01|HV108_HUMAN	Immunoglobulin heavy variable 1-8 (IGHV1-8)	0.01	0.64
36	sp|P56962|STX17_HUMAN	Syntaxin-17 (STX17)	0.00	0.69
37	sp|P09601|HMOX1_HUMAN	Heme oxygenase 1 (HMOX1)	0.02	0.75
38	sp|A0A075B6X5|TVA18_HUMAN	T cell receptor alpha variable 18 (TRAV18)	0.00	0.66
39	sp|P10643|CO7_HUMAN	Complement component C7 (C7)	0.00	0.79
40	sp|Q03933|HSF2_HUMAN	Heat shock factor protein 2 (HSF2)	0.00	0.64
41	sp|A0A0C4DH38|HV551_HUMAN	Immunoglobulin heavy variable 5-51 (IGHV5-51)	0.03	0.78
42	sp|Q15139|KPCD1_HUMAN	Serine/threonine-protein kinase D1 (PRKD1)	0.00	0.81
43	sp|Q9H1X3|DJC25_HUMAN	DnaJ homolog subfamily C member 25 (DNAJC25)	0.00	0.6
44	sp|A4UGR9|XIRP2_HUMAN	Xin actin-binding repeat-containing protein 2 (XIRP2)	0.00	0.68
45	sp|Q8N6N6|NATD1_HUMAN	Protein NATD1 (NATD1)	0.00	0.76
46	sp|A0PJZ3|GXLT2_HUMAN	Glucoside xylosyltransferase 2 (GXYLT2)	0.00	0.81
47	sp|P15169|CBPN_HUMAN	Carboxypeptidase N catalytic chain (CPN1)	0.02	0.71
48	sp|O94952|FBX21_HUMAN	F-box only protein 21 (FBXO21)	0.00	0.83
49	sp|Q4U2R6|RM51_HUMAN	39S ribosomal protein L51, mitochondrial (MRPL51)	0.02	0.83
50	sp|P50749|RASF2_HUMAN	Ras association domain-containing protein 2 (RASSF2)	0.02	0.82
51	sp|Q66PJ3|AR6P4_HUMAN	ADP-ribosylation factor-like protein 6-interacting protein 4 (ARL6IP4)	0.01	0.8
52	sp|O94868|FCSD2_HUMAN	F-BAR and double SH3 domains protein 2 (FCHSD2)	0.03	0.7
53	sp|Q9Y5U8|MPC1_HUMAN	Mitochondrial pyruvate carrier 1 (MPC1)	0.00	0.75
54	sp|Q96NT0|CC115_HUMAN	Coiled-coil domain-containing protein 115 (CCDC115)	0.01	0.78
55	sp|Q9UGJ0|AAKG2_HUMAN	5′-AMP-activated protein kinase subunit gamma-2 (PRKAG2)	0.00	0.81
56	sp|Q0P641|CB080_HUMAN	Uncharacterized protein C2orf80 (C2orf80)	0.00	0.69
57	sp|Q96GM8|TOE1_HUMAN	Target of EGR1 protein 1 (TOE1)	0.01	0.8
58	sp|P01825|HV459_HUMAN	Immunoglobulin heavy variable 4-59 (IGHV4-59)	0.02	0.78
59	sp|Q9BSB4|ATGA1_HUMAN	Autophagy-related protein 101 (ATG101)	0.04	0.81
60	sp|Q53FV1|ORML2_HUMAN	ORM1-like protein 2 (ORMDL2)	0.03	0.81
61	sp|P20742|PZP_HUMAN	Pregnancy zone protein (PZP)	0.00	0.8
62	sp|O15213|WDR46_HUMAN	WD repeat-containing protein 46 (WDR46)	0.01	0.83
63	sp|Q9P1P5|TAAR2_HUMAN	Trace amine-associated receptor 2 (TAAR2)	0.00	0.72
64	sp|P0CG29|GST2_HUMAN	Glutathione S-transferase theta-2 (GSTT2)	0.01	0.75
65	sp|O96028|NSD2_HUMAN	Histone-lysine N-methyltransferase NSD2 (NSD2)	0.05	0.82
66	sp|Q9NX36|DJC28_HUMAN	DnaJ homolog subfamily C member 28 (DNAJC28)	0.00	0.7
67	sp|Q9GZT4|SRR_HUMAN	Serine racemase (SRR)	0.03	0.81
68	sp|Q9NYQ3|HAOX2_HUMAN	Hydroxyacid oxidase 2 (HAO2)	0.00	0.72
69	sp|A2RTX5|SYTC2_HUMAN	Probable threonine–tRNA ligase 2, cytoplasmic (TARSL2)	0.00	0.77
70	sp|P30453|1A34_HUMAN	HLA class I histocompatibility antigen, A-34 alpha chain (HLA-A)	0.02	0.74
71	sp|P78332|RBM6_HUMAN	RNA-binding protein 6 (RBM6)	0.02	0.83
72	sp|P01743|HV146_HUMAN	Immunoglobulin heavy variable 1-46 (IGHV1-46)	0.00	0.8
73	sp|Q8NG11|TSN14_HUMAN	Tetraspanin-14 (TSPAN14)	0.01	0.82
74	sp|Q8TBP5|F174A_HUMAN	Membrane protein FAM174A (FAM174A)	0.01	0.6
75	sp|O60551|NMT2_HUMAN	Glycylpeptide N-tetradecanoyltransferase 2 (NMT2)	0.01	0.81
76	sp|Q99829|CPNE1_HUMAN	Copine-1 (CPNE1)	0.00	0.83
77	sp|Q9Y6A4|CFA20_HUMAN	Cilia- and flagella-associated protein 20 (CFAP20)	0.00	0.79
78	sp|Q8NBF1|GLIS1_HUMAN	Zinc finger protein GLIS1 (GLIS1)	0.05	0.72
79	sp|Q9BQ75|CMS1_HUMAN	Protein CMSS1 (CMSS1)	0.00	0.65
80	sp|O15055|PER2_HUMAN	Period circadian protein homolog 2 (PER2)	0.00	0.69
81	sp|Q96QE5|TEFM_HUMAN	Transcription elongation factor, mitochondrial (TEFM)	0.01	0.61
82	sp|P04114|APOB_HUMAN	Apolipoprotein B-100 (APOB)	0.05	0.83
83	sp|Q8IYA8|IHO1_HUMAN	Interactor of HORMAD1 protein 1 (CCDC36)	0.02	0.7
84	sp|P08571|CD14_HUMAN	Monocyte differentiation antigen CD14 (CD14)	0.00	0.82
85	sp|Q96EV8|DTBP1_HUMAN	Dysbindin (DTNBP1)	0.02	0.76
86	sp|Q15166|PON3_HUMAN	Serum paraoxonase/lactonase 3 (PON3)	0.01	0.82
87	sp|Q8IV63|VRK3_HUMAN	Inactive serine/threonine-protein kinase VRK3 (VRK3)	0.04	0.83
88	sp|P01009|A1AT_HUMAN	Alpha-1-antitrypsin (SERPINA1)	0.02	0.82
89	sp|Q15022|SUZ12_HUMAN	Polycomb protein SUZ12 (SUZ12)	0.00	0.7
90	sp|P30711|GSTT1_HUMAN	Glutathione S-transferase theta-1 (GSTT1)	0.01	0.69
91	sp|Q0VDG4|SCRN3_HUMAN	Secernin-3 (SCRN3)	0.00	0.75
92	sp|P35443|TSP4_HUMAN	Thrombospondin-4 (THBS4)	0.00	0.68
93	sp|Q14680|MELK_HUMAN	Maternal embryonic leucine zipper kinase (MELK)	0.00	0.62
94	sp|Q6DD87|ZN787_HUMAN	Zinc finger protein 787 (ZNF787)	0.00	0.83
95	sp|P00488|F13A_HUMAN	Coagulation factor XIII A chain (F13A1)	0.03	0.81
96	sp|P01766|HV313_HUMAN	Immunoglobulin heavy variable 3-13 (IGHV3-13)	0.01	0.78
97	sp|Q9Y3D7|TIM16_HUMAN	Mitochondrial import inner membrane translocase subunit TIM16 (PAM16)	0.02	0.83
98	sp|Q15843|NEDD8_HUMAN	NEDD8 (NEDD8)	0.02	0.73
99	sp|P02533|K1C14_HUMAN	Keratin, type I cytoskeletal 14 (KRT14)	0.01	0.79
100	sp|Q5UCC4|EMC10_HUMAN	ER membrane protein complex subunit 10 (EMC10)	0.00	0.8
101	sp|O95258|UCP5_HUMAN	Brain mitochondrial carrier protein 1 (SLC25A14)	0.03	0.8
102	sp|Q96Q15|SMG1_HUMAN	Serine/threonine-protein kinase SMG1 (SMG1)	0.01	0.78
103	sp|Q8N5I9|CL045_HUMAN	Uncharacterized protein C12orf45 (C12orf45)	0.00	0.75
104	sp|P51157|RAB28_HUMAN	Ras-related protein Rab-28 (RAB28)	0.02	0.83
105	sp|P27037|AVR2A_HUMAN	Activin receptor type-2A (ACVR2A)	0.05	0.78
106	sp|Q9BT92|TCHP_HUMAN	Trichoplein keratin filament-binding protein (TCHP)	0.04	0.8
107	sp|Q15427|SF3B4_HUMAN	Splicing factor 3B subunit 4 (SF3B4)	0.04	0.82
108	sp|P24593|IBP5_HUMAN	Insulin-like growth factor-binding protein 5 (IGFBP5)	0.05	0.78
109	sp|Q9Y2Z9|COQ6_HUMAN	Ubiquinone biosynthesis monooxygenase COQ6, mitochondrial (COQ6)	0.02	0.8
110	sp|P14136|GFAP_HUMAN	Glial fibrillary acidic protein (GFAP)	0.03	0.75
111	sp|Q8NCG5|CHST4_HUMAN	Carbohydrate sulfotransferase 4 (CHST4)	0.00	0.8
112	sp|P01834|IGKC_HUMAN	Immunoglobulin kappa constant (IGKC)	0.05	0.83
113	sp|Q4ZHG4|FNDC1_HUMAN	Fibronectin type III domain-containing protein 1 (FNDC1)	0.00	0.77
114	sp|Q15653|IKBB_HUMAN	NF-kappa-B inhibitor beta (NFKBIB)	0.04	0.82
115	sp|E7ETH6|Z587B_HUMAN	Zinc finger protein 587B (ZNF587B)	0.05	0.83
116	sp|P49184|DNSL1_HUMAN	Deoxyribonuclease-1-like 1 (DNASE1L1)	0.00	0.66
117	sp|Q96HJ9|FMC1_HUMAN	Protein FMC1 homolog (FMC1)	0.04	0.82
118	sp|Q96BN8|OTUL_HUMAN	Ubiquitin thioesterase otulin (OTULIN)	0.00	0.82
119	sp|Q9HBI5|CC014_HUMAN	Uncharacterized protein C3orf14 (C3orf14)	0.01	0.8
120	sp|O95801|TTC4_HUMAN	Tetratricopeptide repeat protein 4 (TTC4)	0.02	0.83
121	sp|Q9BS92|NPS3B_HUMAN	Protein NipSnap homolog 3B (NIPSNAP3B)	0.01	0.81
122	sp|A0A075B6S2|KVD29_HUMAN	Immunoglobulin kappa variable 2D-29 (IGKV2D-29)	0.02	0.82
123	sp|A0A0C4DH31|HV118_HUMAN	Immunoglobulin heavy variable 1-18 (IGHV1-18)	0.00	0.71
124	sp|Q969X6|UTP4_HUMAN	U3 small nucleolar RNA-associated protein 4 homolog (UTP4)	0.02	0.81
126	sp|Q9NVU7|SDA1_HUMAN	Protein SDA1 homolog (SDAD1)	0.02	1.22
127	sp|Q9BZL1|UBL5_HUMAN	Ubiquitin-like protein 5 (UBL5)	0.02	1.35
128	sp|Q96K80|ZC3HA_HUMAN	Zinc finger CCCH domain-containing protein 10 (ZC3H10)	0.00	1.24
129	sp|O00479|HMGN4_HUMAN	High mobility group nucleosome-binding domain-containing protein 4 (HMGN4)	0.04	1.31
130	sp|P35558|PCKGC_HUMAN	Phosphoenolpyruvate carboxykinase, cytosolic [GTP] (PCK1)	0.04	1.21
131	sp|Q3MIR4|CC50B_HUMAN	Cell cycle control protein 50B (TMEM30B)	0.01	2.09
132	sp|Q53RD9|FBLN7_HUMAN	Fibulin-7 (FBLN7)	0.02	1.34
133	sp|P60983|GMFB_HUMAN	Glia maturation factor beta (GMFB)	0.00	1.22
134	sp|Q15041|AR6P1_HUMAN	ADP-ribosylation factor-like protein 6-interacting protein 1 (ARL6IP1)	0.00	1.52
135	sp|Q9BYX7|ACTBM_HUMAN	Putative beta-actin-like protein 3 (POTEKP)	0.00	1.21
136	sp|Q9HC07|TM165_HUMAN	Transmembrane protein 165 (TMEM165)	0.01	1.27
137	sp|Q9H9Y6|RPA2_HUMAN	DNA-directed RNA polymerase I subunit RPA2 (POLR1B)	0.05	1.27
138	sp|Q8IUF8|RIOX2_HUMAN	Ribosomal oxygenase 2 (RIOX2)	0.00	1.73
139	sp|Q92833|JARD2_HUMAN	Protein Jumonji (JARID2)	0.04	1.25
140	sp|Q587I9|SFT2C_HUMAN	Vesicle transport protein SFT2C (SFT2D3)	0.01	1.25
141	sp|P13498|CY24A_HUMAN	Cytochrome b-245 light chain (CYBA)	0.01	1.28
142	sp|Q58EX2|SDK2_HUMAN	Protein sidekick-2 (SDK2)	0.00	1.3
143	sp|P52823|STC1_HUMAN	Stanniocalcin-1 (STC1)	0.02	2
144	sp|Q9H4B7|TBB1_HUMAN	Tubulin beta-1 chain (TUBB1)	0.04	1.25
145	sp|Q9HA82|CERS4_HUMAN	Ceramide synthase 4 (CERS4)	0.02	1.88
146	sp|Q12866|MERTK_HUMAN	Tyrosine-protein kinase Mer (MERTK)	0.01	1.28
147	sp|Q8IZV5|RDH10_HUMAN	Retinol dehydrogenase 10 (RDH10)	0.01	1.76
148	sp|O15014|ZN609_HUMAN	Zinc finger protein 609 (ZNF609)	0.00	1.26
149	sp|P60468|SC61B_HUMAN	Protein transport protein Sec61 subunit beta (SEC61B)	0.02	1.22
150	sp|Q96AA3|RFT1_HUMAN	Protein RFT1 homolog (RFT1)	0.01	1.31
151	sp|Q6PHR2|ULK3_HUMAN	Serine/threonine-protein kinase ULK3 (ULK3)	0.01	3.02
152	sp|Q5BJF2|SGMR2_HUMAN	Sigma intracellular receptor 2 (TMEM97)	0.01	1.4
153	sp|P63267|ACTH_HUMAN	Actin, gamma-enteric smooth muscle (ACTG2)	0.00	1.37
154	sp|P78563|RED1_HUMAN	Double-stranded RNA-specific editase 1 (ADARB1)	0.03	1.28
155	sp|P16422|EPCAM_HUMAN	Epithelial cell adhesion molecule (EPCAM)	0.01	1.24
156	sp|P31371|FGF9_HUMAN	Fibroblast growth factor 9 (FGF9)	0.01	1.23
157	sp|P57053|H2BFS_HUMAN	Histone H2B type F-S (H2BFS)	0.00	1.27
158	sp|Q9HBR0|S38AA_HUMAN	Putative sodium-coupled neutral amino acid transporter 10 (SLC38A10)	0.04	1.56
159	sp|Q5W0Z9|ZDH20_HUMAN	Palmitoyltransferase ZDHHC20 (ZDHHC20)	0.00	1.29
160	sp|P16112|PGCA_HUMAN	Aggrecan core protein (ACAN)	0.04	1.22
161	sp|Q9H9S4|CB39L_HUMAN	Calcium-binding protein 39-like (CAB39L)	0.00	1.25
162	sp|Q9BWE0|REPI1_HUMAN	Replication initiator 1 (REPIN1)	0.01	1.28
163	sp|Q9GZU7|CTDS1_HUMAN	Carboxy-terminal domain RNA polymerase II polypeptide A small phosphatase 1	0.00	1.26
164	sp|P42680|TEC_HUMAN	Tyrosine-protein kinase Tec (TEC)	0.05	1.24
165	sp|Q9BRI3|ZNT2_HUMAN	Zinc transporter 2 (SLC30A2)	0.04	1.36
166	sp|O14653|GOSR2_HUMAN	Golgi SNAP receptor complex member 2 (GOSR2)	0.02	1.25
167	sp|O75665|OFD1_HUMAN	Oral-facial-digital syndrome 1 protein (OFD1)	0.04	1.52
168	sp|Q14687|GSE1_HUMAN	Genetic suppressor element 1 (GSE1)	0.00	1.21
169	sp|Q9BPX3|CND3_HUMAN	Condensin complex subunit 3 (NCAPG)	0.04	1.36
170	sp|Q96NY8|NECT4_HUMAN	Nectin-4 (NECTIN4)	0.00	1.21
171	sp|Q07507|DERM_HUMAN	Dermatopontin (DPT0)	0.01	1.5
172	sp|P61956|SUMO2_HUMAN	Small ubiquitin-related modifier 2 (SUMO2)	0.02	1.22
173	sp|Q9BZ67|FRMD8_HUMAN	FERM domain-containing protein 8 (FRMD8)	0.00	1.22
174	sp|Q9Y624|JAM1_HUMAN	Junctional adhesion molecule A (F11R)	0.00	1.26
175	sp|P30486|1B48_HUMAN	HLA class I histocompatibility antigen, B-48 alpha chain (HLA-B)	0.01	2.04
176	sp|Q13601|KRR1_HUMAN	KRR1 small subunit processome component homolog (KRR1)	0.00	1.21
177	sp|P27987|IP3KB_HUMAN	Inositol-trisphosphate 3-kinase B (ITPKB)	0.00	1.22
178	sp|P15151|PVR_HUMAN	Poliovirus receptor (PVR)	0.00	1.21
179	sp|O14925|TIM23_HUMAN	Mitochondrial import inner membrane translocase subunit Tim23 (TIMM23)	0.00	1.34
180	sp|Q8N556|AFAP1_HUMAN	Actin filament-associated protein 1 (AFAP1)	0.02	1.3
181	sp|Q9Y3C1|NOP16_HUMAN	Nucleolar protein 16 (NOP16)	0.00	1.33
182	sp|P55290|CAD13_HUMAN	Cadherin-13 (CDH13)	0.00	1.32
183	sp|Q96HI0|SENP5_HUMAN	Sentrin-specific protease 5 (SENP5)	0.00	1.4
184	sp|Q9ULJ3|ZBT21_HUMAN	Zinc finger and BTB domain-containing protein 21 (ZBTB21)	0.01	2.55
185	sp|P27487|DPP4_HUMAN	Dipeptidyl peptidase 4 (DPP4)	0.03	1.49
186	sp|Q8NH19|O10AG_HUMAN	Olfactory receptor 10AG1 (OR10AG1)	0.02	2.06
187	sp|P15309|PPAP_HUMAN	Prostatic acid phosphatase (ACPP)	0.00	1.34
188	sp|Q9ULR0|ISY1_HUMAN	Pre-mRNA-splicing factor ISY1 homolog (ISY1)	0.00	2.08
189	sp|Q96M86|DNHD1_HUMAN	Dynein heavy chain domain-containing protein 1 (DNHD1)	0.02	1.55
190	sp|P0CW20|LIMS4_HUMAN	LIM and senescent cell antigen-like-containing domain protein 4 (LIMS4)	0.01	1.27
191	sp|O75503|CLN5_HUMAN	Ceroid-lipofuscinosis neuronal protein 5 (CLN5)	0.03	1.67
192	sp|Q9HC36|MRM3_HUMAN	rRNA methyltransferase 3, mitochondrial (MRM3)	0.00	1.22
193	sp|Q9H910|JUPI2_HUMAN	Jupiter microtubule associated homolog 2 (JPT2)	0.00	1.25
194	sp|P00414|COX3_HUMAN	Cytochrome c oxidase subunit 3 (MT-CO3)	0.00	1.31
195	sp|Q96EC8|YIPF6_HUMAN	Protein YIPF6 (YIPF6)	0.02	1.31
196	sp|P81605|DCD_HUMAN	Dermcidin (DCD)	0.00	1.24
197	sp|P05423|RPC4_HUMAN	DNA-directed RNA polymerase III subunit RPC4 (POLR3D)	0.01	1.91
198	sp|Q16186|ADRM1_HUMAN	Proteasomal ubiquitin receptor ADRM1 (ADRM1)	0.03	1.28
199	sp|Q86WQ0|NR2CA_HUMAN	Nuclear receptor 2C2-associated protein (NR2C2AP)	0.00	1.24
200	sp|P35527|K1C9_HUMAN	Keratin, type I cytoskeletal 9 (KRT9)	0.00	1.26
201	sp|P05114|HMGN1_HUMAN	Non-histone chromosomal protein HMG-14 (HMGN1)	0.04	1.29
202	sp|P06307|CCKN_HUMAN	Cholecystokinin (CCK)	0.01	1.31
203	sp|Q9UKL6|PPCT_HUMAN	Phosphatidylcholine transfer protein (PCTP)	0.02	1.27
204	sp|Q5T5N4|CF118_HUMAN	Uncharacterized protein C6orf118 (C6orf118)	0.01	1.99
205	sp|Q56VL3|OCAD2_HUMAN	OCIA domain-containing protein 2 (OCIAD2)	0.01	1.21
206	sp|P10109|ADX_HUMAN	Adrenodoxin, mitochondrial (FDX1)	0.03	1.23
207	sp|P62306|RUXF_HUMAN	Small nuclear ribonucleoprotein F (SNRPF)	0.01	1.22
208	sp|Q9P0S3|ORML1_HUMAN	ORM1-like protein 1 (ORMDL1)	0.01	1.22
209	sp|Q9H4K7|MTG2_HUMAN	Mitochondrial ribosome-associated GTPase 2 (MTG2)	0.00	1.24
210	sp|Q8WXI4|ACO11_HUMAN	Acyl-coenzyme A thioesterase 11 (ACOT11)	0.01	1.46
211	sp|Q96JH8|RADIL_HUMAN	Ras-associating and dilute domain-containing protein (RADIL)	0.01	1.22
212	sp|Q9BPU6|DPYL5_HUMAN	Dihydropyrimidinase-related protein 5 (DPYSL5)	0.02	1.21
213	sp|Q9H300|PARL_HUMAN	Presenilins-associated rhomboid-like protein, mitochondrial (PARL)	0.01	1.44
214	sp|Q9NXH9|TRM1_HUMAN	tRNA (guanine(26)-N(2))-dimethyltransferase (TRMT1)	0.04	1.22
215	sp|Q6UUV7|CRTC3_HUMAN	CREB-regulated transcription coactivator 3 (CRTC3)	0.00	1.74

### Functional classification of differentially expressed proteins (DEPs) in the endometrium

To determine the functional differences in the increased and decreased proteins, the quantified proteins were analyzed for the following three types of enrichment-based clustering analyses: gene ontology (GO) enrichment analysis of DEPs, pathway enrichment analysis of DEPs, and eukaryotic orthologous groups (KOGs) annotation of DEPs.

GO enrichment analysis showed the GO terms in which the DEPs were enriched in all identified proteins. It represented the important or typical biological functions in the study. We performed pathway enrichment analysis of DEPs based on the Kyoto Encyclopedia of Genes and Genomes (KEGG) database. KOGs were delineated by comparing protein sequences encoded in complete genomes, which represented major phylogenetic lineages.

**Fig. 3:**
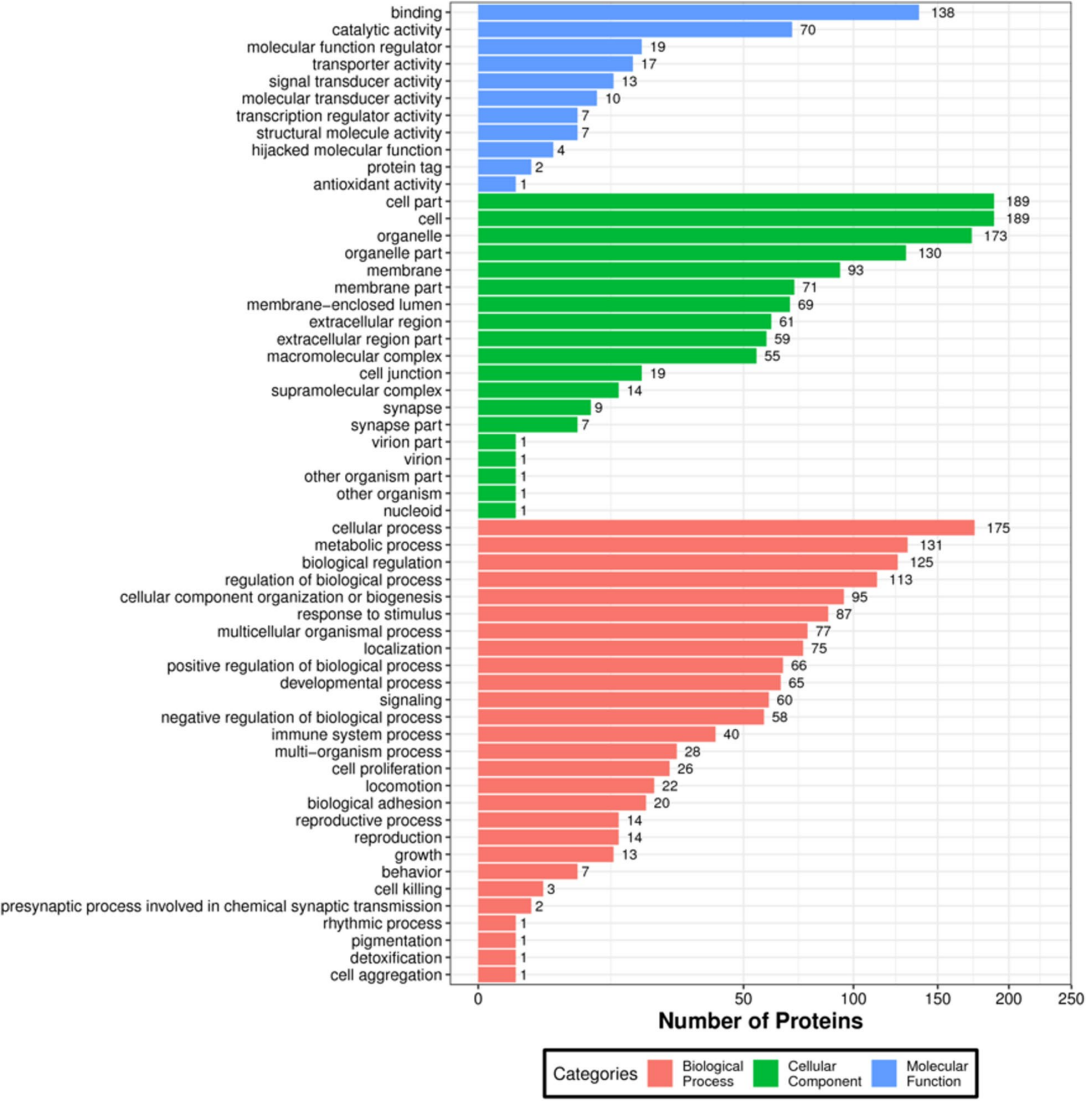
Gene Ontology Analysis of Differentially Expressed Proteins x-axis displays protein count, y-axis displays GO term

Through the GO enrichment analysis of biological processes, we found that these different proteins were closely associated with cellular processes, metabolic processes, and biological regulation. Based on their molecular functions, these proteins with altered levels were strongly associated with binding, catalytic activity, and molecular function regulators (Fig. 3, Additional file 1: Fig. S2).

**Fig. 4:**
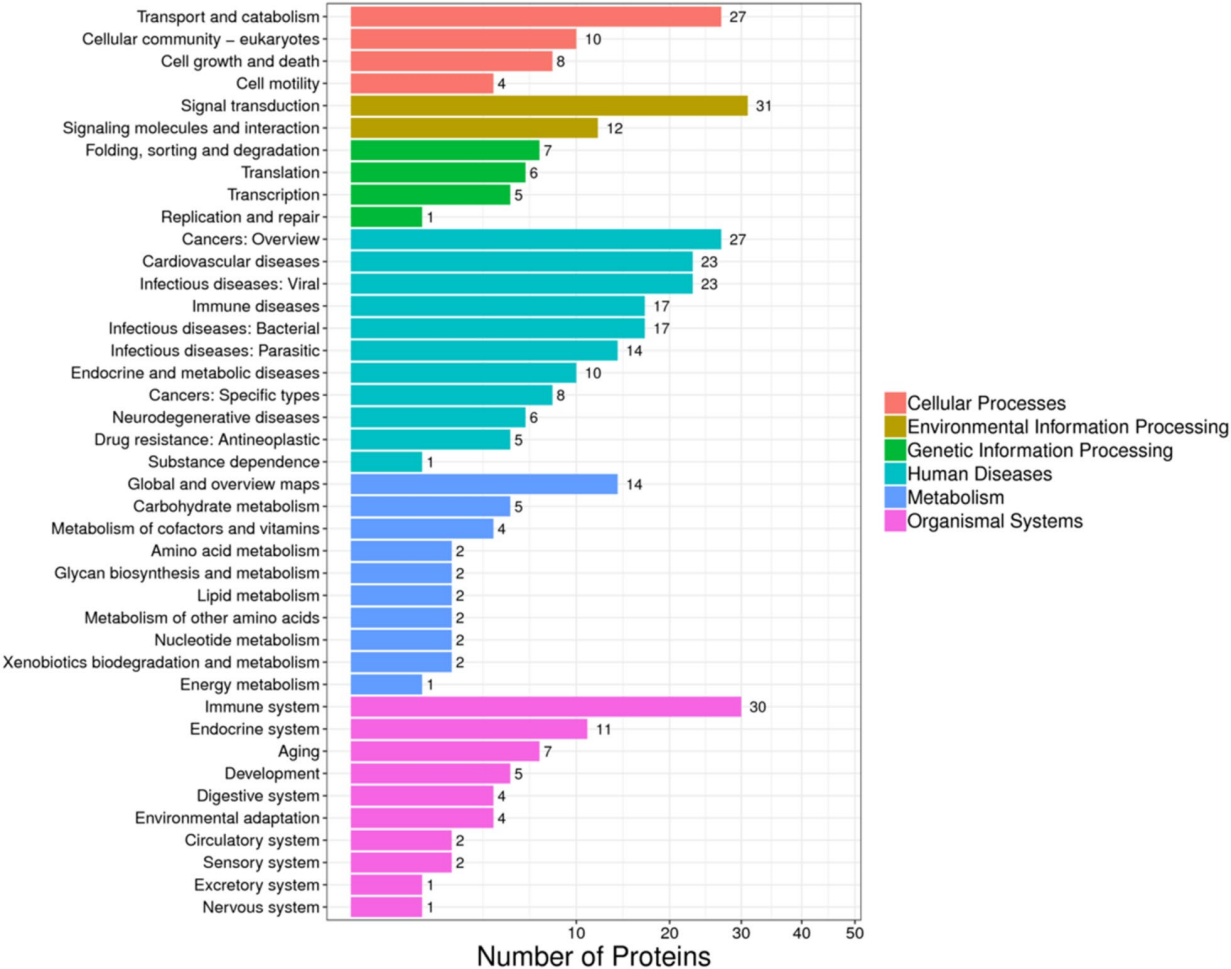
Pathway analysis of differentially expressed proteins

**Fig. 5:**
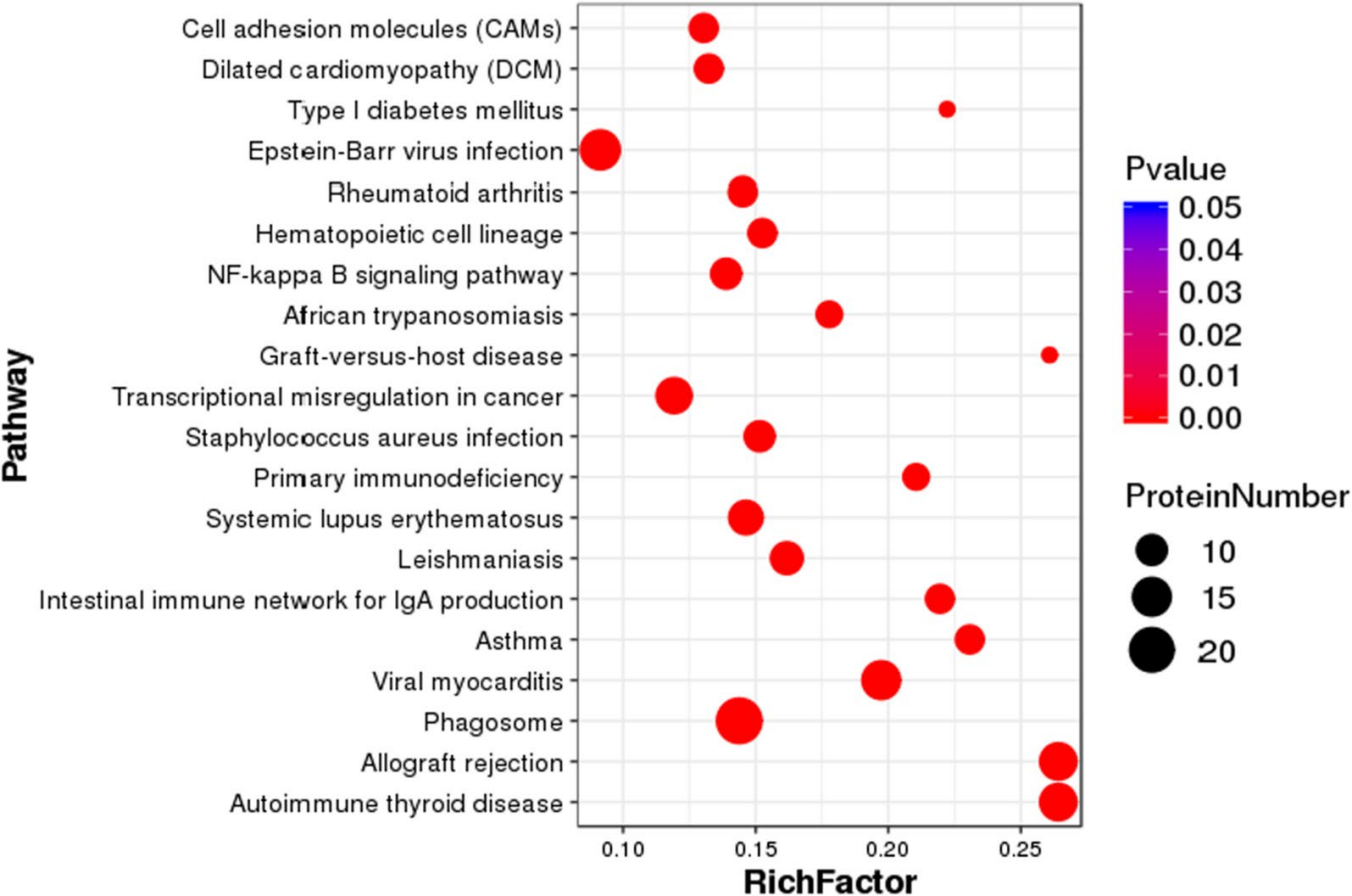
Statistics of pathway enrichment of differentially expressed proteins in each pairwise. RichFactor is the ratio of differentially expressed protein number annotated in this pathway term to all protein number annotated in this pathway term. Greater richFator means greater intensiveness. P value ranges from 0–1, and less P value means greater intensiveness. We just display the top 20 of enriched pathway terms.

The results from KEGG pathway enrichment showed that the DEPs were mainly involved in allograft rejection, cell adhesion molecules (CAMs), type I diabetes mellitus, allograft rejection, phagosomes, and the necrotic factor (NF)—kappa B signaling pathway (Fig. 4, Additional file 1: Fig. S3). Moreover, we constructed a scatter plot for the top 20 KEGG enrichment results as shown in Fig. 5.

**Fig. 6:**
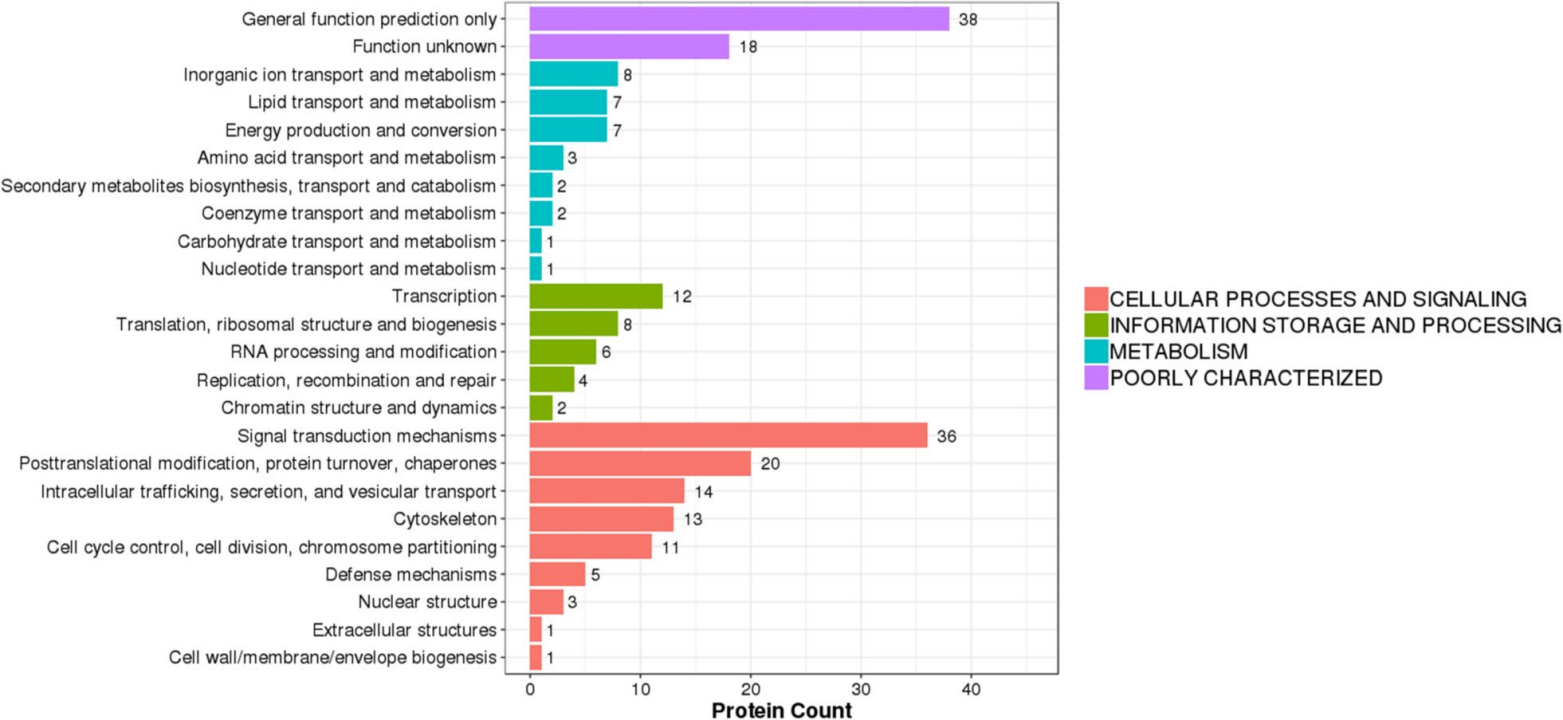
KOG Annotation of differentially expressed proteins

For DEPs, their KOG terms were also extracted and showed that the DEPs were mainly associated with inorganic ion transport and metabolism, lipid transport and metabolism, and energy production and conversion. We plotted bar plots accordingly (Fig. 6). Thus, we could easily obtain their functional categories.

#### Predicted protein–protein interactions (PPI) of DEPs and subcellular localization prediction of DEPs

**Fig. 7:**
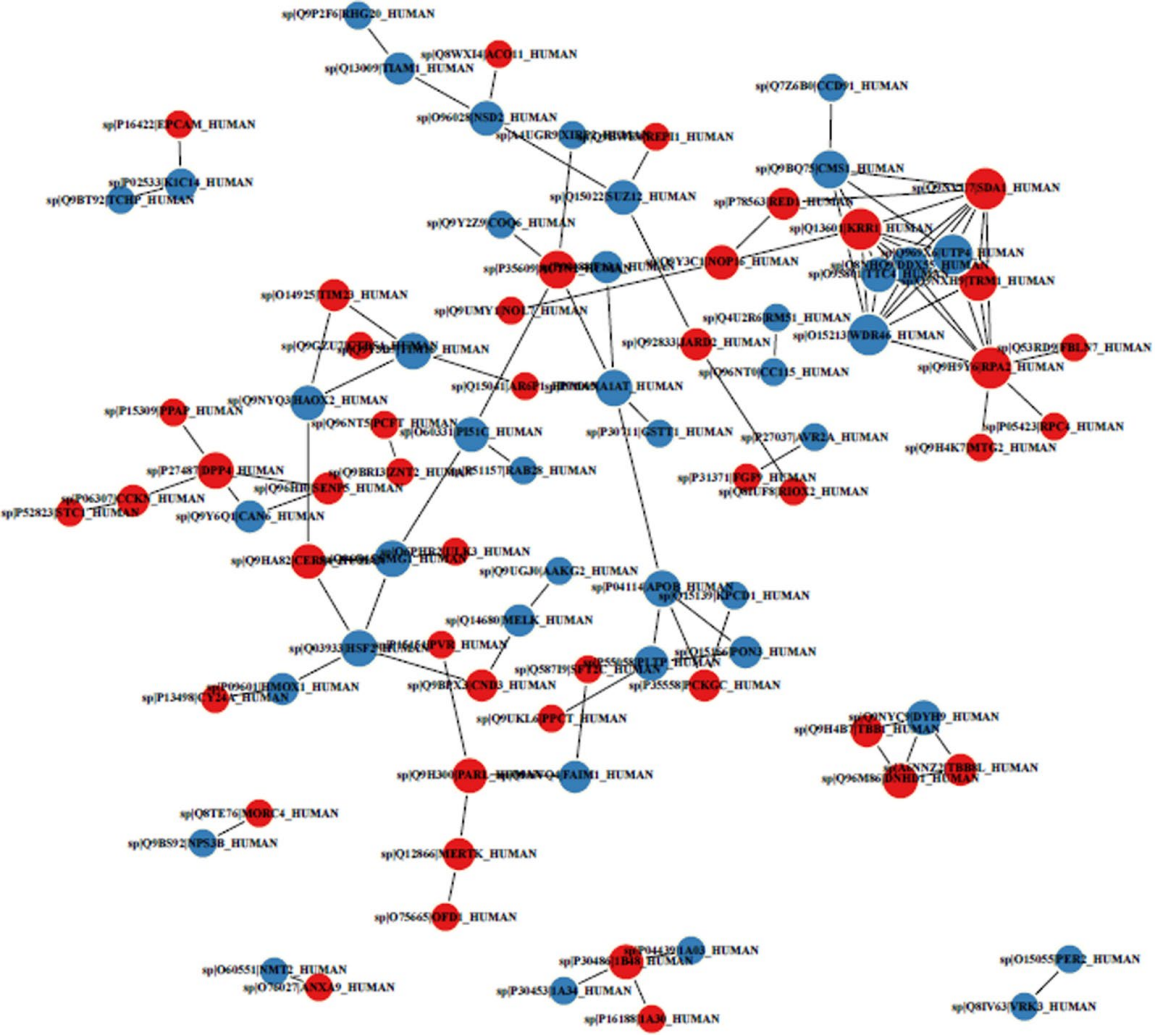
PPI Network of differentially expressed proteins. Red and blue circle represent up-regulated and down-regulated proteins separately. Edges with different colors represent classes of KEGG pathway (Red: Cellular Processes; Blue: Environmental Information Processing; Green: Genetic Information Processing; Purple: Human Diseasea; Orange: Metabolism; Yellow: Organismal Systems; Brown: Drug Development)

**Fig. 8:**
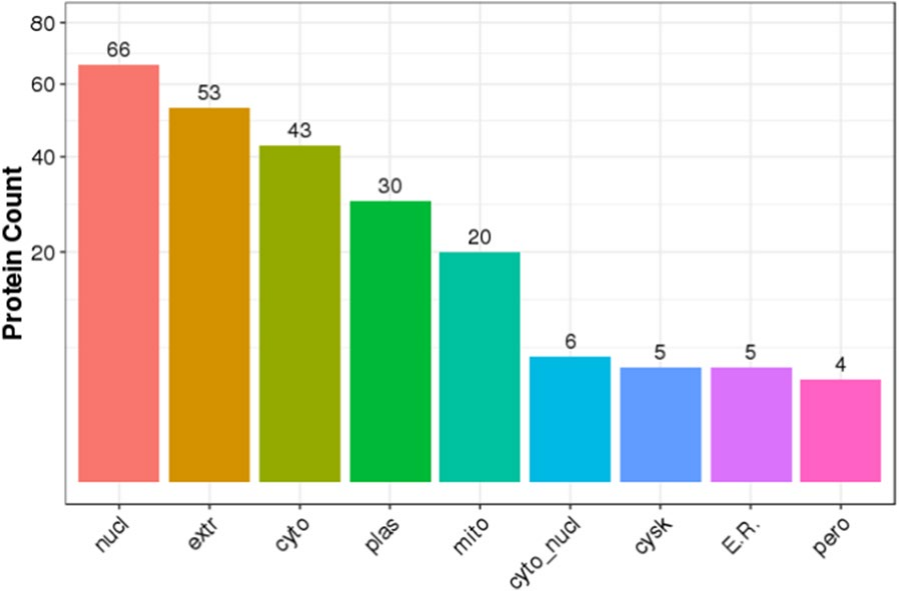
Subcellular localization prediction of differentially expressed proteins x-axis displays subcellular structure; y-axis displays protein count

Proteins usually interact with each other to participate in certain biological functions. STRING is a database of known PPI. Based on Fig. 7, we determined the interaction between proteins (Fig. 7). Proteins can be targeted in the inner space of an organelle, different intracellular membranes, the plasma membrane, or to the exterior of the cell through secretion. This delivery process is performed on the basis of the information present in the protein. Correct sorting is important for the cell; errors can lead to the development of diseases. We predicted protein subcellular localization using bioinformatics tools (WoLF PSORT). The bar plot of subcellular localization prediction showed that different proteins are mainly present in the nucleus, extracellular space, cytosol, plasma membrane, and mitochondria (Fig. 8).

Taken together, these results showed that these DEPs mainly play a role in metabolic processes, cell adhesion molecules, and immunity.

## Discussion

Embryo implantation is a key process in pregnancy. For successful embryo implantation, the process must be sequential, which means that the three phases, namely apposition, adhesion, and invasion, should occur sequentially [16]. For pregnancy, endometrium transition to the pregnancy state is the key to embryo implantation, and a change in several proteins in the endometrium during this process is a prerequisite [17, 18]. The DEPs discovered in the present study were mainly involved in energy metabolism, inflammation, and cell–cell adhesion functions, as well as the cell and cell parts in cellular components and catalytic activity. Energy metabolism may affect embryo implantation, whereas inflammation and CAMs may affect both endometrial conversion and receptivity.

### Impairment of embryo implantation because of energy metabolism deficit

The exact mechanism of embryo implantation is not clear, and probably energy metabolism is a crucial factor in implantation [19]. PCOS is an endocrine disorder characterized by hyperinsulinemia and obesity [20]. These characteristics can cause an insulin-resistant state and metabolic disorder in organs such as the endometrium [21, 22]. As insulin resistance in the endometrium leads to no response or sensitivity to the metabolic effects of insulin, the endometrium needs more insulin for normal metabolism [23]. The gene for insulin-like growth factor-binding protein 5 (IGFBP5) is downregulated in patients with PCOS than in healthy people, and IGFBP5 is an important member of the IGFBP family. IGFBP5 may affect cell metabolism. A decrease in IGFBP5 level may be associated with the pathogenesis of type 2 diabetes [24, 25], and decreased GLUT4 expression may be one of the mechanisms by which IGFBP causes insulin resistance [26]. Moreover, the results of our subcellular localization analysis show that many different proteins are located in mitochondria. Importantly, mitochondria play a key role in energy production by converting nutrients into energy, and altered proteins may negatively affect energy metabolism. For example, mitochondrial pyruvate carrier 1 (MPC1) and transcription elongation factor mitochondrial (TEFM) levels were significantly decreased in patients with PCOS. Pyruvate, carried by MPC1 into the mitochondrion, is essential to mitochondrial energy metabolism. The lack of MPC1 can impair pyruvate transport and then can damage mitochondrial energy metabolism [27]. The final site of glucose metabolism is in mitochondria, in which TEFM regulates the formation of mitochondrial RNA primers. As RNA primers are necessary for the initiation of mitochondrial DNA replication, the lack of TEFM reduces mitochondrial DNA replication [28]. Therefore, abnormalities in MPC1 and TEFM must affect mitochondrial oxidation, thus leading to a bioenergetic crisis. Therefore, we hypothesized that energy metabolism deficits may cause embryo implant failure, and treatment including energy supplements may improve the endometrial microenvironment.

### CAM deficiency causes miscarriage

Apart from energy metabolism deficits, embryo implantation also requires adhesion molecules. Increasing or decreasing adhesion molecules can lead to embryo implantation failure. In our proteomics analysis results, we observed the differential expression of adhesion molecules in the PCOS group including CAMs, receptor–ligand activity, and cell adhesion. Among these, epithelial CAM (EpCAM) level was increased in endometrial samples of women with PCOS. EpCAM regulates many important cellular functions such as cell migration, metastasis, proliferation, and cell differentiation [29, 30]; however, the main role of EpCAM is intercellular adhesion [31]. A specific EpCAM is necessary for embryo implantation, and the amount of EpCAM during the implantation window should be reduced [32]. EpCAMs are maintained mainly at the basal cell surface to maintain a polarized epithelial surface, and then uterine epithelial cells connect with the underlying stroma to prevent premature detachment before implantation [33]. However, higher concentrations of EpCAM can impair adhesion or promote deadhesion by competitively binding to extracellular matrix proteins and blocking cell attachment. Proteomics analysis results show that T-lymphoma invasion and metastasis-inducing protein 1 (TIAM1) were decreased in the PCOS group, which regulates cell migration, motility, and cell adhesion in some cells [34, 35]. TIAM1 is decreased by estradiol and increased by progesterone in a dose-dependent manner [36]. Patients with PCOS lack a complete menstrual cycle as a result of oligo- or anovulation; thus, the endometrium is exposed to estradiol for an extended period and lacks progesterone [37]. The reduction in TIMA1 level is consistent with the characteristics of patients with PCOS. TIAM1 is essential in embryo implantation in mice by increasing the implantation site of the endometrium [38]. Studies have shown that increased levels of TIAM1 during the implantation window facilitates embryo implantation, and decreased TIAM1 levels might be associated with the failure of embryo implantation in patients with repeated implantation failure [35]. More studies need to be established to explore the details of adhesion mechanisms underlying the endometrium of PCOS.

### Immune disorders lead to miscarriage

The embryo is a natural semi-allograft, and tolerance mechanisms for successful embryo implantation involve the acceptance of allografts [39]. A recent study highlighted that immune imbalance plays a key role in recurrent miscarriage [40]. Our pathway analysis reports that allograft rejection, natural killer (NK)-cell-mediated cytotoxicity, and primary immunodeficiency in patients with PCOS were significantly abnormal compared with those in healthy women. For instance, human leukocyte antigen C (HLA-C), a marker of recurrent miscarriage, was significantly increased in the PCOS group [41]. In the fetal–maternal interface, NK cells recognize and eliminate exogenous cells mainly resulting from HLA expressed on the foreign cell surface [42]. Thus, the increased HLA-C levels may negatively affect the process by which NK cells recognize embryo antigens, resulting in immune tolerance disorders. Hemeoxygenase 1 (HMOX1) was significantly decreased in patients with PCOS. HMOX1 is a central player in anti-inflammatory, antioxidant, and cytoprotective activities, and HMOX1 can inhibit the cytotoxicity of other immune cells, cytokine release, and proliferation [43, 44]. HMOX1 is necessary for protecting fetuses from rejection [45, 46]. Therefore, HMOX1 deficiency may affect fetal and allograft rejection, thereby leading to embryo implantation failure. Thus, curing immune disorders in the endometrium will improve the probability of embryo implantation success.

### Strengths and limitations of the study

Our results show that endometrial receptive damage in patients with PCOS is not only associated with a single factor but also multiple proteins, pathways, systems, and other abnormalities; these factors also interact with each other. Due to difficulty in obtaining the desired endometrial tissues repeatedly at the same time, we only compared endometrial proteomics in the luteal phase between the experimental group and the control group, rather than comparing the endometrial proteomics in different phases in one group. Moreover, animal validation model tests are in preparation.

## Conclusion

Our results show that endometrial receptive damage in patients with PCOS is not a single factor event but occurs because of multiple proteins, pathways, systems, and other abnormalities, and they also interact with each other, thereby greatly increasing the difficulty of endometrial receptive research. More studies are needed to support the hypothesis of this study and to establish a better understanding of the molecular mechanistic details underlying impaired endometrial implantation in patients with PCOS.

## Supplementary Information

**Additional file 1**: **Fig. S1.** CV distribution in replicate. **Fig. S2.** Gene Ontology Analysis of Differentially Expressed Proteins. **Fig. S3.** Pathway analysis of Differentially Expressed Proteins.

## Data Availability

The mass spectrometry proteomics data have been deposited to the ProteomeXchange Consortium via the PRIDE [47] partner repository with the dataset identifier PXD024735.
