# Supplementary material for: Proteomic alteration of endometrial tissues during secretion in polycystic ovary syndrome may affect endometrial receptivity
Source: Clin Proteomics. 2022 May 28;19:19. doi: 10.1186/s12014-022-09353-1 (PMC9145147; doi:10.1186/s12014-022-09353-1)
Supplement: Supplementary file 1 — Additional file 1: Fig. S1. CV distribution in replicate. Fig. S2. Gene Ontology Analysis of Differentially Expressed Proteins. Fig. S3. Pathway analysis of Differentially Expressed Proteins. [file 12014_2022_9353_MOESM1_ESM.docx]

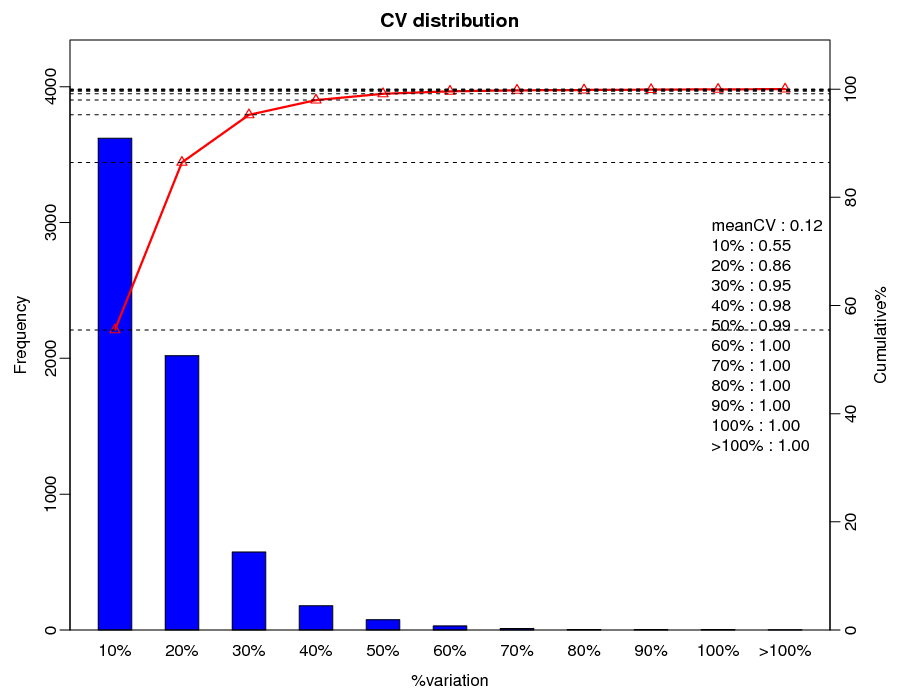


**Fig. S1 CV distribution in replicate.**

X-axis is the deviation between the protein ratio of the repetitive samples. Y-axis is the percentage that protein at a certain angle comprise quantified protein amout.


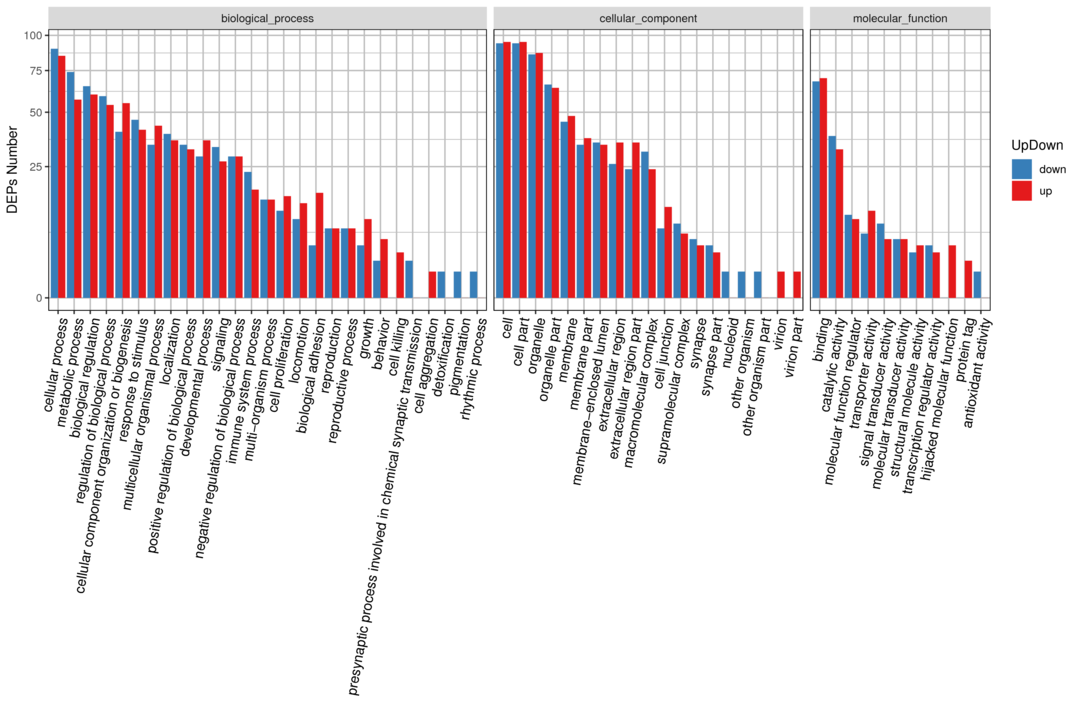


**Fig. S2. Gene Ontology Analysis of Differentially Expressed Proteins.**

**(**x-axis displays GO term，y axis displays protein count.**)**


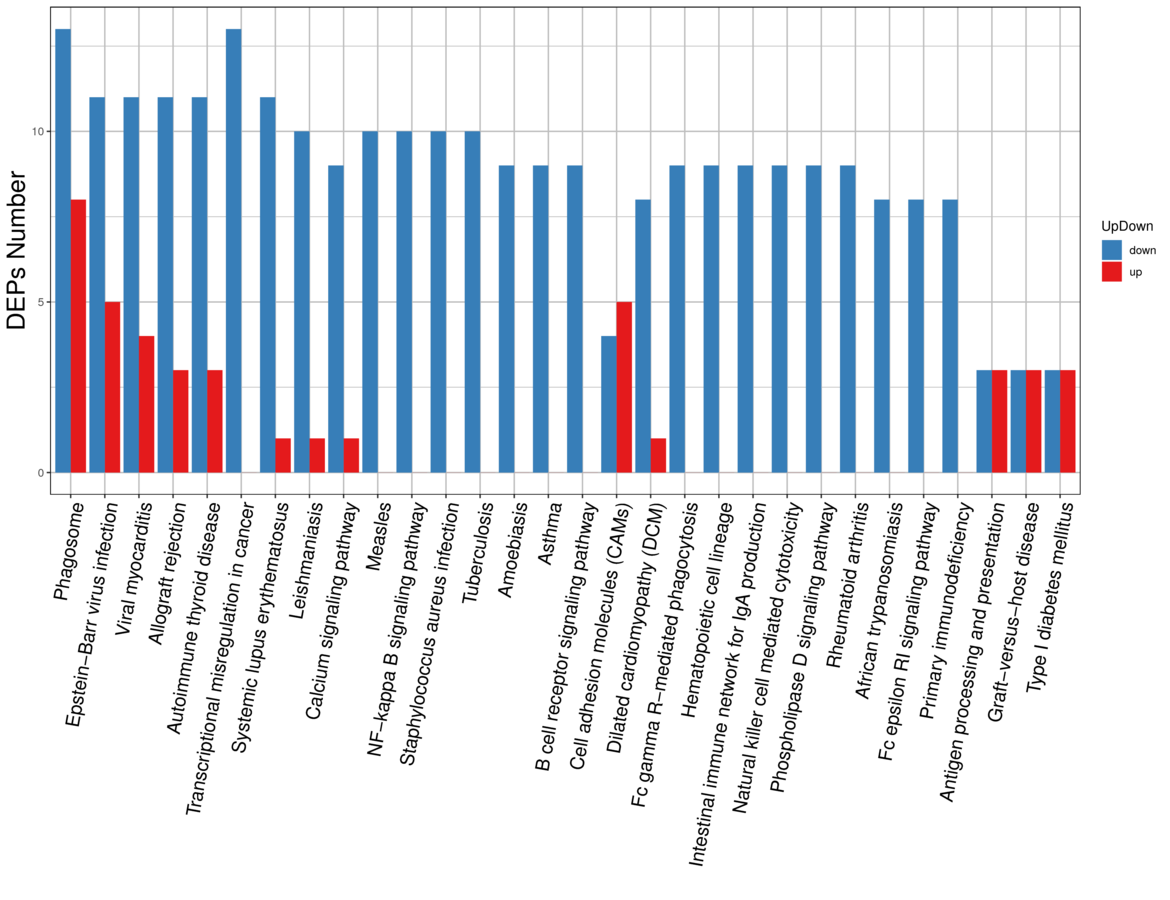


**Fig. S3. Pathway analysis of Differentially Expressed Proteins.**

（x-axis displays pathway name, y-axis displays differentially expressed protein count.）
